# Supplementary material for: Applying a systems thinking approach to evaluating the effectiveness of Africa’s foodborne disease surveillance systems
Source: Commun Med (Lond). 2025 Sep 25;5:397. doi: 10.1038/s43856-025-01115-y (PMC12462454; doi:10.1038/s43856-025-01115-y)
Supplement: Supplementary file 2 — Supplementary Information [file 43856_2025_1115_MOESM2_ESM.pdf]

Supplementary Material

Thystrup et. al. - Applying a systems thinking approach to evaluating the effectiveness of Africa's foodborne disease surveillance systems

Supplementary Table 1: List of questions used to guide the workshop for problem structuring in Arusha, Tanzania, February 14-17th 2024.

Layer of the iceberg	Questions to address the layer of the iceberg
Events	What is currently happening with foodborne diseases in African low- and middle-income countries?
Patterns/trends	What patterns and trends do we observe?
Underlying structures	What are influencing these patterns?
Mental models	What assumptions, beliefs and values do people hold about foodborne diseases and the current foodborne disease surveillance system in an African context?

Supplementary Figure 1: A causal loop diagram (CLD) titled "Public trust and surveillance reporting" showing the balancing loop of how public mistrust can lead to reduced compliance with surveillance systems and reporting, thus increasing the public awareness and incidence of FBD. Arrows indicate directional relationships between elements: red arrows signify negative or decreasing effects, while blue arrows indicate positive or increasing effects.

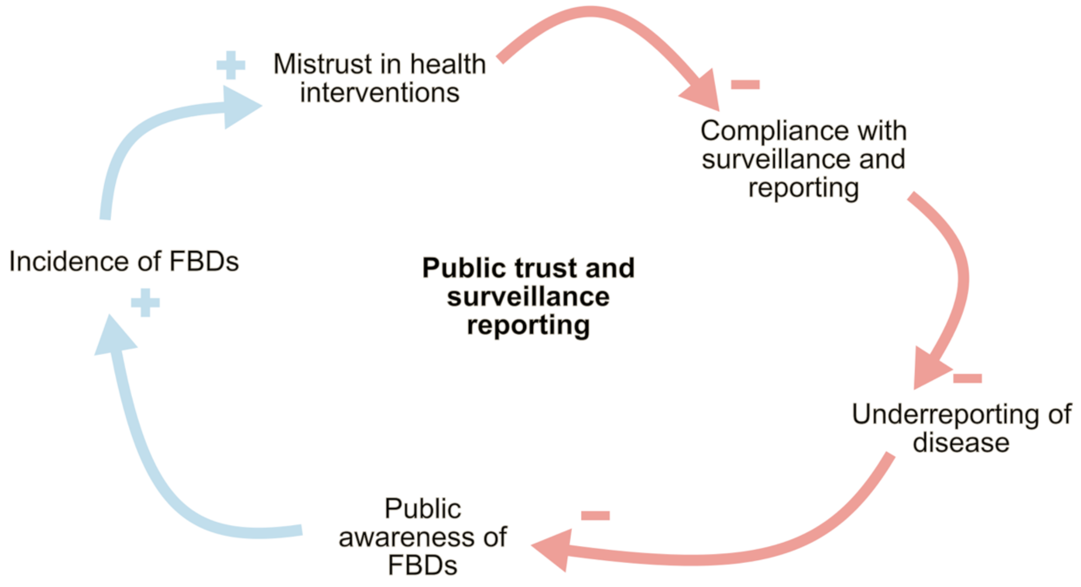

Supplementary Figure 2: A causal loop diagram (CLD) titled "Education and cost-benefit perception of food safety" showing the reinforcing loop of how persistently limited education will reduce the understanding of food safety's importance, thus leading to normalization of illness, and reduced cost-benefit of food safety, which leads to lower investment in surveillance and protection. Arrows indicate directional relationships between elements: red arrows signify negative or decreasing effects, while blue arrows indicate positive or increasing effects.

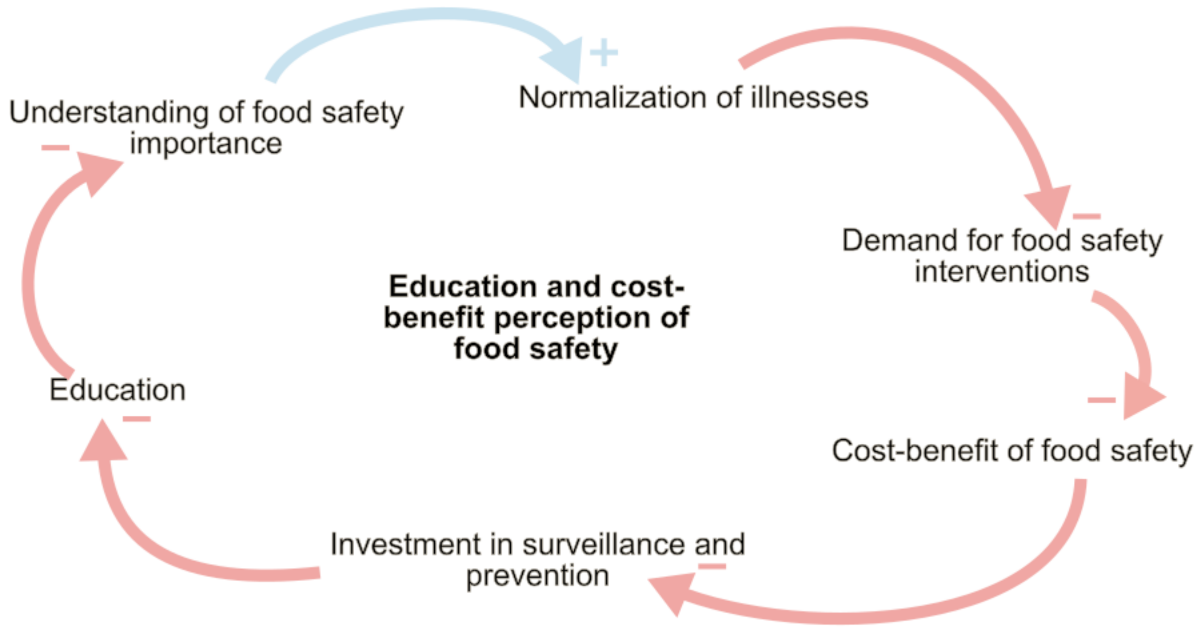

Supplementary Figure 3: A causal loop diagram (CLD) titled "Public pressure and system response" showing the balancing loop of how increased incidence of FBDs can create public pressure for system improvements, which then grows and peaks with high incidence levels, leading to minor improvements. The implementation of surveillance will then gradually reduce FBD incidence and stabilize the system. Arrows indicate directional relationships between elements: red arrows signify negative or decreasing effects, while blue arrows indicate positive or increasing effects.

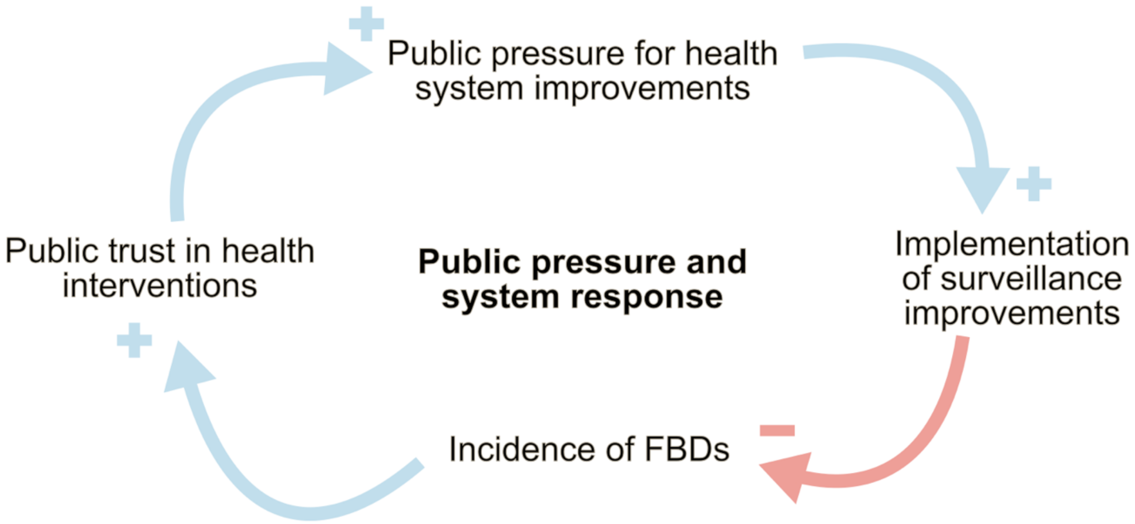

Supplementary Figure 4: A causal loop diagram (CLD) titled "Diagnostic capacity and reporting of disease" showing the reinforcing loop of how diagnostic capacity decreases over time, due to lack of resources and limited reporting, which will lead to underreporting of disease and gradual increase of FBD incidence. Arrows indicate directional relationships between elements: red arrows signify negative or decreasing effects, while blue arrows indicate positive or increasing effects.

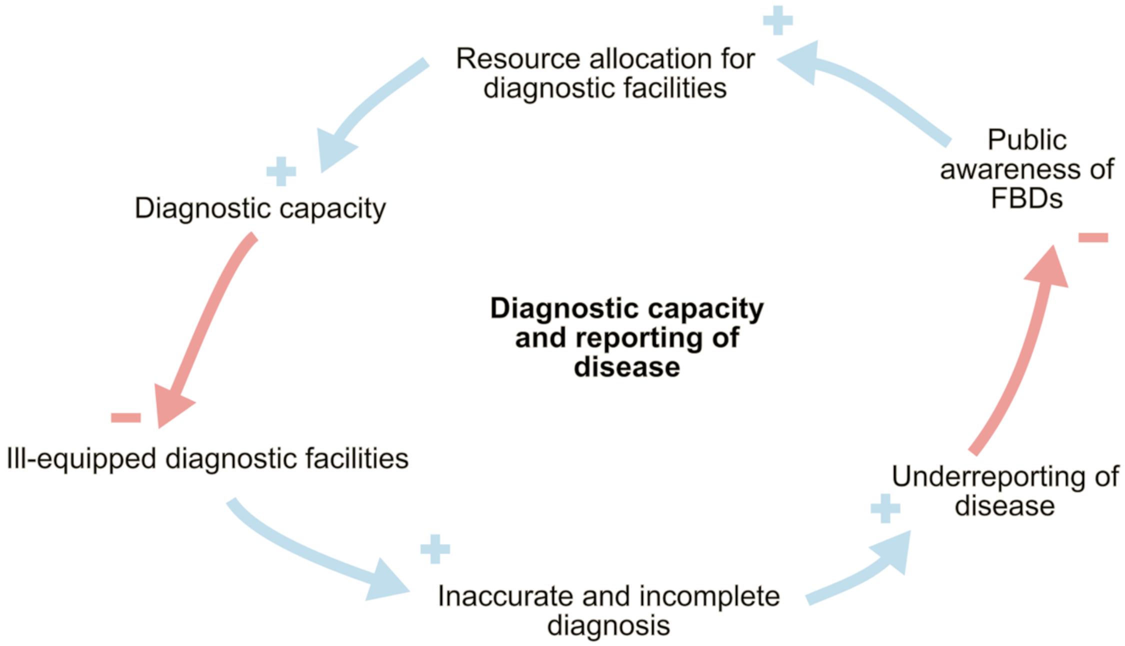

Supplementary Figure 5: A causal loop diagram (CLD) titled "Severity of illness and resource strain" showing the reinforcing loop of how increased severity of illness can lead to more hospital admissions and resource strain, decreasing the funding for surveillance over time. Arrows indicate directional relationships between elements: red arrows signify negative or decreasing effects, while blue arrows indicate positive or increasing effects.

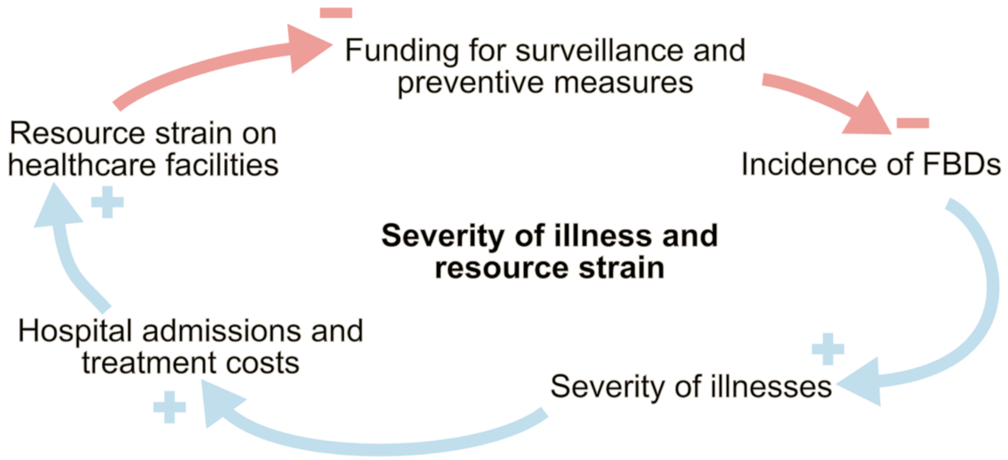

Supplementary Figure 6: A causal loop diagram (CLD) titled "Food production and safety compliance" showing the reinforcing loop of how economic pressure on the food production can lead to increased use of antimicrobials, thus resulting in a decreased effectiveness of medical treatment for FBDs. Arrows indicate directional relationships between elements: red arrows signify negative or decreasing effects, while blue arrows indicate positive or increasing effects.

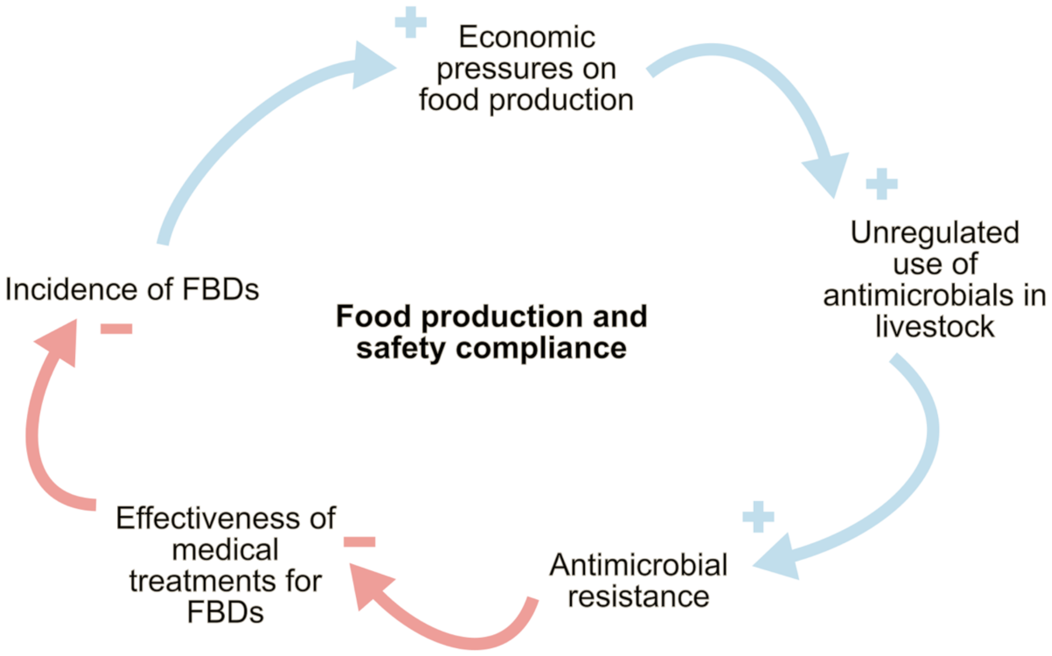

Supplementary Figure 7: A causal loop diagram (CLD) titled "Cross-contamination cycle in agriculture" showing the reinforcing loop of how the compliance with proper food handling can lead to increased incidence of FBDs through cross-contamination. Arrows indicate directional relationships between elements: red arrows signify negative or decreasing effects, while blue arrows indicate positive or increasing effects.

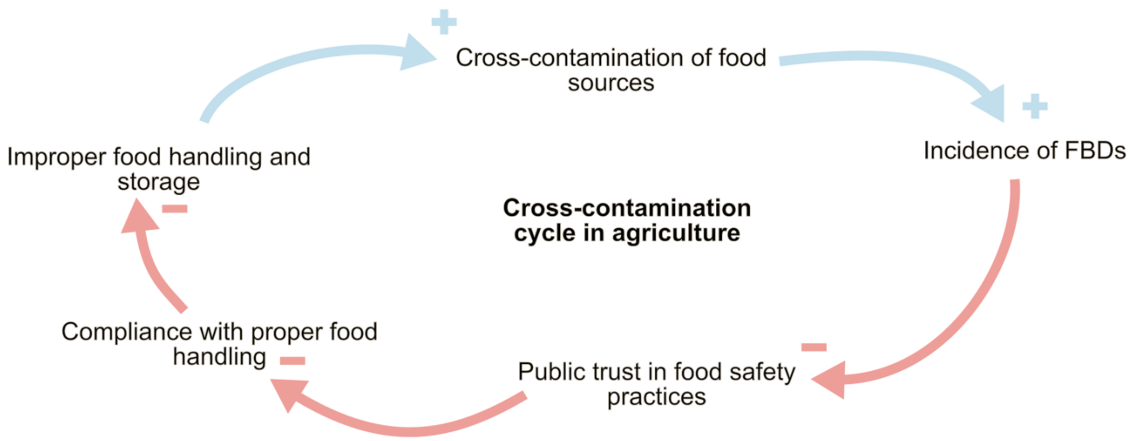

Supplementary Figure 8: A causal loop diagram (CLD) titled "Public demand and agricultural surveillance" showing the balancing loop of how public demand for safe food practices can lead directly to increased incidence of FBDs. Arrows indicate directional relationships between elements: red arrows signify negative or decreasing effects, while blue arrows indicate positive or increasing effects.

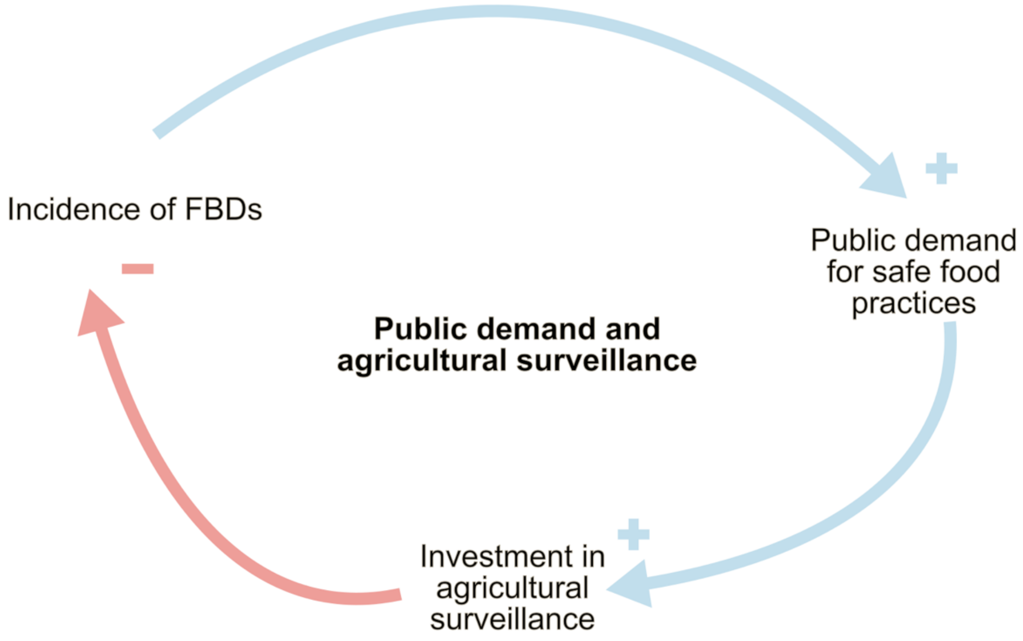

Supplementary References

1. Ho-Palma, A. C. *et al.* Salmonella in Chicken and Pork Meat as a Likely Major Contributor to Foodborne Illness in Peru. *Am J Trop Med Hyg* **111**, 141–150 (2024).
2. BALOGH de, K., HALLIDAY, J. & LUBROTH, J. Integrating the surveillance of animal health, foodborne pathogens and foodborne diseases in developing and in-transition countries. *Revue Scientifique et Technique de l'OIE* **32**, 539–548 (2013).
3. Ombui, J. N., Kagiko, M. M. & Arimi, S. M. Foodborne diseases in Kenya. *East Afr Med J* **78**, (2001).
4. Asuming-Bediako, N., Parry-Hanson Kunadu, A., Abraham, S. & Habib, I. Campylobacter at the Human–Food Interface: The African Perspective. *Pathogens* **8**, 87 (2019).
5. Kirk, M. D. *et al.* World Health Organization Estimates of the Global and Regional Disease Burden of 22 Foodborne Bacterial, Protozoal, and Viral Diseases, 2010: A Data Synthesis. *PLoS Med* **12**, (2015).
6. Havelaar, A. H. *et al.* World Health Organization Global Estimates and Regional Comparisons of the Burden of Foodborne Disease in 2010. *PLoS Med* **12**, e1001923 (2015).
7. Cissé, G. Food-borne and water-borne diseases under climate change in low- and middle-income countries: Further efforts needed for reducing environmental health exposure risks. *Acta Trop* **194**, 181–188 (2019).
8. Thystrup, C. *et al.* Etiology-specific incidence and mortality of diarrheal diseases in the African region: a systematic review and meta-analysis. *BMC Public Health* **24**, 1864 (2024).
9. Seyoum, E. T. *et al.* Pre-Harvest Food Safety Challenges in Food-Animal Production in Low- and Middle-Income Countries. *Animals* **14**, 786 (2024).
10. Imade, F. *et al.* Updates on food and feed mycotoxin contamination and safety in Africa with special reference to Nigeria. *Mycology* **12**, 245–260 (2021).
11. *Stronger Food and Drug Regulatory Systems Abroad*. (National Academies Press, Washington, D.C., 2020). doi:10.17226/25651.
12. Garcia, S. N., Mpatswenumugabo, J. P. M., Ntampaka, P., Nandi, S. & Cullor, J. S. A one health framework to advance food safety and security: An on-farm case study in the Rwandan dairy sector. *One Health* **16**, 100531 (2023).
13. Mwape, R. K., Barday, M.-A., van der Zalm, M. M. & Verhagen, L. M. Overview of mucosal immunity and respiratory infections in children: a focus on Africa. *Curr Opin Pediatr* **37**, 137–144 (2025).
14. Bates, M., Marais, B. J. & Zumla, A. Tuberculosis Comorbidity with Communicable and Noncommunicable Diseases. *Cold Spring Harb Perspect Med* **5**, a017889 (2015).
15. Mutasa, K. *et al.* Stunting Status and Exposure to Infection and Inflammation in Early Life Shape Antibacterial Immune Cell Function Among Zimbabwean Children. *Front Immunol* **13**, (2022).
16. Yapi, H. F. *et al.* Déficit en fer, profil protéique immunitaire, inflammatoire et nutritionnel chez l'enfant de Côte-d'Ivoire. *Cahiers de Santé* **19**, 25–28 (2009).

17. Ammoun, R. *et al.* Readiness of health facilities to deliver non-communicable diseases services in Kenya: a national cross-sectional survey. *BMC Health Serv Res* **22**, 985 (2022).
18. Obiero, C. W. *et al.* Clinical features to distinguish meningitis among young infants at a rural Kenyan hospital. *Arch Dis Child* **106**, 130–136 (2021).
19. Musinguzi, G. *et al.* Capacity of Health Facilities to Manage Hypertension in Mukono and Buikwe Districts in Uganda: Challenges and Recommendations. *PLoS One* **10**, e0142312 (2015).
20. Mbituyumuremyi, A. *et al.* Controlling hepatitis C in Rwanda: a framework for a national response. *Bull World Health Organ* **96**, 51–58 (2018).
21. Daniel, A. I. *et al.* Biofertilizer: The Future of Food Security and Food Safety. *Microorganisms* **10**, 1220 (2022).
22. Tibebu, A., Tamrat, H. & Bahiru, A. Review: Impact of food safety on global trade. *Vet Med Sci* **10**, (2024).
23. Boggess, M. V. *et al.* The need for agriculture phenotyping: "Moving from genotype to phenotype". *J Proteomics* **93**, 20–39 (2013).
24. Gonçalves, D. da C., Ribeiro, W. R., Gonçalves, D. C., Menini, L. & Costa, H. Recent advances and future perspective of essential oils in control Colletotrichum spp.: A sustainable alternative in postharvest treatment of fruits. *Food Research International* **150**, 110758 (2021).
25. Dhollander, S. *et al.* A Systematic Literature Review of Variables Associated with the Occurrence of African Swine Fever. *Viruses* **17**, 192 (2025).
26. Razavi-Shearer, D. *et al.* Global prevalence, cascade of care, and prophylaxis coverage of hepatitis B in 2022: a modelling study. *Lancet Gastroenterol Hepatol* **8**, 879–907 (2023).
27. Bunge, E. M. *et al.* The changing epidemiology of human monkeypox—A potential threat? A systematic review. *PLoS Negl Trop Dis* **16**, e0010141 (2022).
28. Kaakoush, N. O., Castaño-Rodríguez, N., Mitchell, H. M. & Man, S. M. Global Epidemiology of Campylobacter Infection. *Clin Microbiol Rev* **28**, 687–720 (2015).
29. Brundisini, F. *et al.* Chronic disease patients' experiences with accessing health care in rural and remote areas: a systematic review and qualitative meta-synthesis. *Ont Health Technol Assess Ser* **13**, 1–33 (2013).
30. Abdalla, O. & Woods, C. Barriers and potential solutions to improve access and equity in prenatal screening for rural women. *Aust N Z J Obstet Gynaecol* **61**, E22–E23 (2021).
31. Marcin, J. P., Shaikh, U. & Steinhorn, R. H. Addressing health disparities in rural communities using telehealth. *Pediatr Res* **79**, 169–176 (2016).
32. Brusnahan, A., Carrasco-Tenezaca, M., Bates, B. R., Roche, R. & Grijalva, M. J. Identifying health care access barriers in southern rural Ecuador. *Int J Equity Health* **21**, 55 (2022).
33. Gizaw, Z. Public health risks related to food safety issues in the food market: a systematic literature review. *Environ Health Prev Med* **24**, 68 (2019).
34. Manning, L. & Soon, J. M. Food Safety, Food Fraud, and Food Defense: A Fast Evolving Literature. *J Food Sci* **81**, (2016).
35. Djekic, I. & Smigic, N. Consumer Perception of Food Fraud in Serbia and Montenegro. *Foods* **13**, 53 (2023).

36. Onyeaka, H., Kalane, M. S., Guta, A. T. & Tamasiga, P. Food fraud amid COVID-19 in Sub-Saharan Africa: A challenge of the present. *Public Health in Practice* **3**, 100234 (2022).
37. Grace, D. Food Safety in Low and Middle Income Countries. *Int J Environ Res Public Health* **12**, 10490–10507 (2015).
38. Masters, W. A., Martinez, E. M., Greb, F., Herforth, A. & Hendriks, S. L. The Cost and Affordability of Preparing a Basic Meal Around the World. in *Science and Innovations for Food Systems Transformation* 603–623 (Springer International Publishing, Cham, 2023). doi:10.1007/978-3-031-15703-5_33.
39. Njoagwuani, E. I. *et al.* Food safety in vulnerable populations: A perspective on the challenges and solutions. *The FASEB Journal* **37**, (2023).
40. Waage, J. *et al.* Changing food systems and infectious disease risks in low-income and middle-income countries. *Lancet Planet Health* **6**, e760–e768 (2022).
41. Threlfall, E. J. Antimicrobial drug resistance in *Salmonella* : problems and perspectives in food- and water-borne infections. *FEMS Microbiol Rev* **26**, 141–148 (2002).
42. Matle, I., Mbatha, K. R. & Madoroba, E. A review of Listeria monocytogenes from meat and meat products: Epidemiology, virulence factors, antimicrobial resistance and diagnosis. *Onderstepoort Journal of Veterinary Research* **87**, (2020).
43. Samtiya, M., Matthews, K. R., Dhewa, T. & Puniya, A. K. Antimicrobial Resistance in the Food Chain: Trends, Mechanisms, Pathways, and Possible Regulation Strategies. *Foods* **11**, 2966 (2022).
44. Mdegela, R. H. *et al.* Antimicrobial Use, Residues, Resistance and Governance in the Food and Agriculture Sectors, Tanzania. *Antibiotics* **10**, 454 (2021).
45. Mason, J. B. & Mitchell, J. T. Nutritional surveillance. *Bull World Health Organ* **61**, 745–55 (1983).
46. Refaya, A. K. *et al.* A review on bovine tuberculosis in India. *Tuberculosis* **122**, 101923 (2020).
47. Hartinger, S. M. *et al.* The 2023 Latin America report of the Lancet Countdown on health and climate change: the imperative for health-centred climate-resilient development. *The Lancet Regional Health - Americas* **33**, 100746 (2024).
48. de Macedo Couto, R., Santana, G. O., Ranzani, O. T. & Waldman, E. A. One Health and surveillance of zoonotic tuberculosis in selected low-income, middle-income and high-income countries: A systematic review. *PLoS Negl Trop Dis* **16**, e0010428 (2022).
49. Mirón, I. J., Linares, C. & Díaz, J. The influence of climate change on food production and food safety. *Environ Res* **216**, 114674 (2023).
50. Watts, N. *et al.* The Lancet Countdown: tracking progress on health and climate change. *The Lancet* **389**, 1151–1164 (2017).
51. Lamar, F. *et al.* Accumulation of microbial hazards and assessment of food hygiene associated with broiler chicken processing at open air food markets in Maputo, Mozambique. *Int J Food Microbiol* **427**, 110960 (2025).
52. Ferreira Rodrigues, J. *et al.* Effect of the COVID-19 pandemic on food habits and perceptions: A study with Brazilians. *Trends Food Sci Technol* **116**, 992–1001 (2021).
53. Utaaker, K. S., Kumar, A., Joshi, H., Chaudhary, S. & Robertson, L. J. Checking the detail in retail: Occurrence of Cryptosporidium and Giardia on vegetables sold across different counters in Chandigarh, India. *Int J Food Microbiol* **263**, 1–8 (2017).

54. Pouillot, R. *et al.* A Risk Assessment of Campylobacteriosis and Salmonellosis Linked to Chicken Meals Prepared in Households in Dakar, Senegal. *Risk Analysis* **32**, 1798–1819 (2012).
55. Burstein, R. *et al.* Mapping 123 million neonatal, infant and child deaths between 2000 and 2017. *Nature* **574**, 353–358 (2019).
56. Salamandane, A., Malfeito-Ferreira, M. & Brito, L. The Socioeconomic Factors of Street Food Vending in Developing Countries and Its Implications for Public Health: A Systematic Review. *Foods* **12**, 3774 (2023).
57. Manjang, B. *et al.* Promoting hygienic weaning food handling practices through a community-based programme: intervention implementation and baseline characteristics for a cluster randomised controlled trial in rural Gambia. *BMJ Open* **8**, e017573 (2018).
58. Barnett, T. & Fournié, G. Zoonoses and wet markets: beyond technical interventions. *Lancet Planet Health* **5**, e2–e3 (2021).
59. Berry, E. D. & Wells, J. E. Reducing Foodborne Pathogen Persistence and Transmission in Animal Production Environments: Challenges and Opportunities. *Microbiol Spectr* **4**, (2016).
60. Young, I., Greig, J., Wilhelm, B. J. & Waddell, L. A. Effectiveness of Food Handler Training and Education Interventions: A Systematic Review and Meta-Analysis. *J Food Prot* **82**, 1714–1728 (2019).
61. Christiana Cudjoe, D., Balali, G. I., Titus, O. O., Osafo, R. & Taufiq, M. Food Safety in Sub-Sahara Africa, An insight into Ghana and Nigeria. *Environ Health Insights* **16**, 117863022211424 (2022).
62. Wallace, F., Mittal, N., Lambertini, E. & Nordhagen, S. Vendor Knowledge, Attitudes, and Practices Related to Food Safety in Low- and Middle-Income Countries: A Scoping Review. *J Food Prot* **85**, 1069–1078 (2022).
63. Dhudum, B. & Bhosale, Y. Street Food Handlers' Knowledge and Hygiene Practices: A Descriptive Study. *Cureus* (2025) doi:10.7759/cureus.77894.
64. Velavan, T. P. *et al.* Hepatitis E: An update on One Health and clinical medicine. *Liver International* **41**, 1462–1473 (2021).
65. Bacha, T. *et al.* Botulism outbreak in a rural Ethiopia: a case series. *BMC Infect Dis* **21**, 1270 (2021).
66. Téllez, G. *et al.* Food-producing animals and their health in relation to human health. *Microb Ecol Health Dis* **26**, (2015).
67. Njoga, E. O. *et al.* Pre-slaughter, slaughter and post-slaughter practices of slaughterhouse workers in Southeast, Nigeria: Animal welfare, meat quality, food safety and public health implications. *PLoS One* **18**, e0282418 (2023).
68. Csete, J. *et al.* Public health and international drug policy. *The Lancet* **387**, 1427–1480 (2016).
69. Duke, T. *et al.* World Health Organization and knowledge translation in maternal, newborn, child and adolescent health and nutrition. *Arch Dis Child* **107**, 644–649 (2022).
70. Awoyomi, O. J. *et al.* Mpox in Nigeria: Perceptions and knowledge of the disease among critical stakeholders—Global public health consequences. *PLoS One* **18**, e0283571 (2023).

71. Metcalfe, J. J. & Leonard, D. The relationship between culinary skills and eating behaviors: Challenges and opportunities for parents and families. *Physiol Behav* **191**, 95–99 (2018).
72. Ogutu, E. A. *et al.* Determinants of food preparation and hygiene practices among caregivers of children under two in Western Kenya: a formative research study. *BMC Public Health* **22**, 1865 (2022).
73. Gobena, T. *et al.* Foodborne Bacterial Pathogens in Animal, Food, and Environmental Samples Collected From the Physical Exposure of Children With Diarrhea in Ethiopia: A One Health Approach. *Environ Health Insights* **18**, (2024).
74. Berendes, D. M. *et al.* Associations between open drain flooding and pediatric enteric infections in the MAL-ED cohort in a low-income, urban neighborhood in Vellore, India. *BMC Public Health* **19**, 926 (2019).
75. Peng, M., Tabashsum, Z., Millner, P., Parveen, S. & Biswas, D. Influence of Manure Application on the Soil Bacterial Microbiome in Integrated Crop-Livestock Farms in Maryland. *Microorganisms* **9**, 2586 (2021).
76. Kniel, K. E., Kumar, D. & Thakur, S. Understanding the Complexities of Food Safety Using a “One Health” Approach. *Microbiol Spectr* **6**, (2018).
77. Devi, K. R. *et al.* Occupational exposure and challenges in tackling M. bovis at human–animal interface: a narrative review. *Int Arch Occup Environ Health* **94**, 1147–1171 (2021).
78. Cao Ba, K., Kaewkungwal, J., Pacheun, O., Nguyen Thi To, U. & Lawpoolsri, S. Health Literacy Toward Zoonotic Diseases Among Livestock Farmers in Vietnam. *Environ Health Insights* **14**, (2020).
79. Alebie, A. & Tewachew, T. Household Practice Related to Zoonotic Diseases Transmission in Rural Community of Gondar Zuria District. *Veterinary Medicine: Research and Reports* **Volume 12**, 109–115 (2021).
80. Nyokabi, N. S. *et al.* From farm to table: exploring food handling and hygiene practices of meat and milk value chain actors in Ethiopia. *BMC Public Health* **23**, 899 (2023).
81. E Nonga, H., Sells, P. & Karimuribo, E. D. Occurrences of thermophilic Campylobacter in cattle slaughtered at Morogoro municipal abattoir, Tanzania. *Trop Anim Health Prod* **42**, 73–78 (2010).
82. Salam, Md. A. *et al.* Antimicrobial Resistance: A Growing Serious Threat for Global Public Health. *Healthcare* **11**, 1946 (2023).
83. Samreen, Ahmad, I., Malak, H. A. & Abulreesh, H. H. Environmental antimicrobial resistance and its drivers: a potential threat to public health. *J Glob Antimicrob Resist* **27**, 101–111 (2021).
84. Neogi, S. B. *et al.* Risk of multi-drug resistant Campylobacter spp. and residual antimicrobials at poultry farms and live bird markets in Bangladesh. *BMC Infect Dis* **20**, 278 (2020).
85. Alam, M.-U. *et al.* Human exposure to antimicrobial resistance from poultry production: Assessing hygiene and waste-disposal practices in Bangladesh. *Int J Hyg Environ Health* **222**, 1068–1076 (2019).
86. Gebre, G. G., Legesse, T. & Fikadu, A. A. Food safety knowledge, attitude, and practice among male and female food handlers: Evidence from fruit and vegetable producers in Ethiopia. *Heliyon* **9**, e17301 (2023).

87. Azanaw, J., Dagne, H., Andualem, Z. & Adane, T. Food Safety Knowledge, Attitude, and Practice of College Students, Ethiopia, 2019: A Cross-Sectional Study. *Biomed Res Int* **2021**, (2021).
88. Abdilahi, M. M. *et al.* Food safety practice and its associated factors among food handlers working in food and drinking establishments in Hargeisa, Somaliland. *BMC Public Health* **25**, 438 (2025).
89. Parikh, P., Aparo, N. O., Nordhagen, S. & De Steur, H. Food safety-related perspectives and practices of consumers and vendors in Ethiopia: A scoping review. *Food Research International* **157**, 111376 (2022).
90. Fantaye, A. W., Gunawardena, N. & Yaya, S. Preferences for formal and traditional sources of childbirth and postnatal care among women in rural Africa: A systematic review. *PLoS One* **14**, e0222110 (2019).
91. Barro, A. *et al.* Knowledge, beliefs and perceptions of religious leaders on modern contraceptive use in Burkina Faso: a qualitative study. *Pan African Medical Journal* **39**, (2021).
92. Stefanini, A. Influence of Health Education on Local Beliefs. *Trop Doct* **17**, 132–134 (1987).
93. Anthonj, C., Giovannini, P. & Kistemann, T. Coping with ill-health: health care facility, chemist or medicinal plants? Health-seeking behaviour in a Kenyan wetland. *BMC Int Health Hum Rights* **19**, 18 (2019).
94. Bedson, J. *et al.* A review and agenda for integrated disease models including social and behavioural factors. *Nat Hum Behav* **5**, 834–846 (2021).
95. Ssewanyana, D., Mwangala, P. N., van Baar, A., Newton, C. R. & Abubakar, A. Health Risk Behaviour among Adolescents Living with HIV in Sub-Saharan Africa: A Systematic Review and Meta-Analysis. *Biomed Res Int* **2018**, 1–18 (2018).
