# Supplementary material for: Applying a systems thinking approach to evaluating the effectiveness of Africa’s foodborne disease surveillance systems
Source: Commun Med (Lond). 2025 Sep 25;5:397. doi: 10.1038/s43856-025-01115-y (PMC12462454; doi:10.1038/s43856-025-01115-y)
Supplement: Supplementary file 3 — Description of Additional Supplementary files [file 43856_2025_1115_MOESM3_ESM.pdf]

Description of Additional Supplementary files

File name: Supplementary Data 1

Description: Origins and occupation of participants and facilitators in the expert workshop in Arusha, Tanzania, February 14-17th 2024

File name: Supplementary Data 2

Description: Search strategy for scoping review on foodborne disease surveillance challenges.

File name: Supplementary Data 3

Description: Archetypes identified in the CLDs through the "System Archetype Basics: From Story to Structure" methodology.
